# Sleeping Beauties of Coronavirus Research

**DOI:** 10.1109/ACCESS.2021.3052918

**Published:** 2021-01-19

**Authors:** Mohsen Fazeli-Varzaneh, Ali Ghorbi, Marcel Ausloos, Emanuel Sallinger, Sahar Vahdati

**Affiliations:** Department of Knowledge and Information ScienceFaculty of ManagementUniversity of Tehran 48425 Tehran 1417466191 Iran; School of BusinessUniversity of Leicester 4488 Leicester LE2 1RQ U.K.; Department of Statistics and EconometricsBucharest University of Economic Studies 125536 010552 Bucharest Romania; GRAPES 4031 Liege Belgium; Faculty of InformaticsTU Wien 27259 1040 Vienna Austria; Department of Computer ScienceUniversity of Oxford 6396 Oxford OX1 3QD U.K.; Nature-Inspired Machine IntelligenceInstitute of Applied Informatics (InfAI) 01069 Dresden Germany

**Keywords:** Beauty score, bibliometric, coronavirus, COVID-19, sleeping beauty, activity index, relative specialization index

## Abstract

A “Sleeping Beauty” (SB) in science is a metaphor for a scholarly publication that remains relatively unnoticed by the related communities for a long time; – the publication is “sleeping”. However, suddenly due to the appearance of some phenomenon, such a “forgotten” publication may become a center of scientific attention; – the SB is “awakened”. Currently, there are specific scientific areas for which sleeping beauties (SBs) are awakened. For example, as the world is experiencing the COVID-19 global pandemic (triggered by SARS-CoV-2), publications on coronaviruses appear to be awakened. Thus, one can raise questions of scientific interest: are these publications coronavirus related SBs? Moreover, while much literature exists on other coronaviruses, there seems to be no comprehensive investigation on COVID-19, - in particular in the context of SBs. Nowadays, such SB papers can be even used for sustaining literature reviews and/or scientific claims about COVID-19. In our study, in order to pinpoint pertinent SBs, we use the “beauty score” (B-score) measure. The Activity Index (AI) and the Relative Specialization Index (RSI) are also calculated to compare countries where such SBs appear. Results show that most of these SBs were published previously to the present epidemic time (triggered by SARS-CoV or SARS-CoV-1), and are awakened in 2020. Besides outlining the most important SBs, we show from what countries and institutions they originate, and the most prolific author(s) of such SBs. The citation trend of SBs that have the highest B-score is also discussed.

## I. Introduction

Some scholarly articles may never be cited, but others become instant hits, receiving many citations as early as the first few years after their publication. It has been shown that the number of citations of most articles peaks around two to ten years from their publication year, but the peak width and its position depend very much on the research field [1]–[6]. However, there are articles that attract none or only very few citations for a long time. Yet, some papers may suddenly see their citation number bursts [7], [8]. Van Raan [9] was the first to label as **“sleeping beauties”** (SBs), articles with such a delayed recognition. Later on, Braun *et al.* [10] identified as “princes” the (first) citing articles that are highly co-cited with the pertinent SB article, which has a “suddenly recognized value”.

In 2020, the SARS coronavirus 2 (SARS-CoV-2, provisionally known as 2019-nCoV) has been the main topic of many social media channels and has become an intense research field. In the last few months, a plethora of scientific papers have been published about SARS-CoV-2. However, SARS-CoV-2 is only one type of Coronaviruses (CoVs). In fact, much research has already been taking place on such viruses, for a long time. The first notice of CoV may be traced back to 1931 – in North Dakota, USA [11]. Up to recent times, such topics were of some interest but somewhat out of the mainstream research in virology and epidemiology. Yet, due to SARS-CoV-2, “old” research papers on Coronaviruses are reappearing. Are these “sleeping beauties” of Coronavirus research?

The **goal** of this article is to analyze whether such papers are sleeping beauties in Coronavirus research, to debate on their discovery, and to discuss their bibliometric properties. This is **challenging** as it requires uniting knowledge and methods from multiple areas: in a first step, one requires *conceptualization and retrieval* of relevant publications, their *acquisition and integration*, and their *enrichment and curation*. While in the case of COVID-19, many research groups from the field of computer science started to gather relevant publications and built COVID-19 “Knowledge Graphs” [12]–[16], none of such investigations are fully suited for the purpose of discussing SBs so far.

Another challenge is the identification of *relevant metrics*, including those that define sleeping beauties, besides those that allow to analysis publications and their connections in scientific, geographical, or other contexts. A final challenge is the *presentation and interpretation of relevant results* since there is a multitude of perspectives along which the results can be presented.

The **main contributions** of the present paper are:

• An overall **overview of sleeping beauties** in the area of coronaviruses, including citations over time and distribution of “beauty scores”.
• The identification and analysis of **countries, institutions, authors, and venues** according to their role in the sleeping beauties research on coronaviruses. This also includes the analysis of key phrases and keywords in awakened coronavirus sleeping beauties.

Beyond the scientific goals, we hope that our research also has some societal **impact** in helping to answer the following questions:

1) Is it possible to identify sleeping beauties “early”?, - thus, perhaps, accelerating COVID-19 research findings and *in fine* optimizing control of the pandemics?
2) Is there a way for stakeholders (such as countries, institutions, and journals) to understand their role in both short-term and long-term coronavirus research? - in particular regarding research that at first glance does not appear important, but later on turns out to be important.

**Organization.**This article is organized as follows: In Section II, we give a brief literature overview on SBs. In Section III, we give a very brief primer on coronaviruses. The main sections of the paper are Section IV and Section V. In Section IV, we describe our method, including data preparation steps and metrics. In Section V, which is the bulk of the work, we outline the results of our analyses. We conclude the work in Section VI by a discussion, and in Section VII by an outlook.

## II. Brief Literature Review

Sleeping beauties have been reported pointing to authors, journals, research fields, and specific subjects of study. The following examples (somewhat listed in chronological order), taken from different research fields, should not be considered as an exhaustive literature review, but only as illustrations in order to mention research topics. To the best of our knowledge, there was no research on Coronaviruses SBs until now. The paradigm of making some potentially influential publications as sleeping beauties but not considering them as such has been discussed by different communities. It is a domain-independent paradigm that is visible in different scientific communities. Here we mention some reports from different research domains where there is a mention of SBs.

In the medical field, Ohba and Nakao [17] found that sleeping beauties exist in Ophthalmology journals. Gorry and Ragouet [18] found that the paper that led to the birth of Interventional Radiology is a sleeping beauty. Furthermore, Završnik *et al.* [19], in Pediatrics, El Aichouchi and Gorry [20] in Oncology, point to SBs. Ho and Hartley [21] found SBs in Psychology.

In “humanities”, Peirce’s short note in Science in 1884 shows a remarkable increase in citations in recent times: his paper “The Numerical Measure of the Success of Predictors” [22], received less than 1 citation per year in the decades prior to 2000, 3.5 citations per year in the 2000s, and 10.4 per year in the 2010s [23]. One might consider that C.S. Peirce’s work (1839-1914) is (or has been) one of the “oldest” sleeping beauties since his work was awakened in the 1970s, - after about one century of sleep.

Tal and Gordon [24] and Teixeira *et al.* [25] found SBs (and princes) in Political Sciences and International Economics and International Business fields, respectively. Hou and Yang [26] found SBs in Social Media-based research.

In Physical and Engineering Sciences, Marx [27] showed that the Shockley-Queisser paper [28] on p-n junction solar cells is an example of SB. Interestingly, - in particular, within our concern, van Raan [29] indicated that SBs might be a pool from which innovations can arise. This was confirmed by Teixeira *et al.* [30] and El Aichouchi and Gorry [31].

Each of these papers provides a bibliography. More generally, such works conducted on SBs have been reviewed earlier, see, for example, Burrell [32], Li and Ye [33], Li [34]; Zhang and Ye specifically cover cases in physics and economics [35].

## III. Decades of Scientific Research on Corona Viruses

Coronaviruses (CoVs) constitute a group of RNA viruses characterized by spherical, enveloped virus particles with prominent surface projections, resembling the corona of the sun [36]. “Apart from infecting a variety of economically important vertebrates (such as pigs and chickens), six coronaviruses have been known to infect human hosts and cause respiratory diseases. Among them, severe acute respiratory syndrome coronavirus (SARS-CoV) and Middle East respiratory syndrome coronavirus (MERS-CoV) are zoonotic and highly pathogenic coronaviruses that have resulted in regional and global outbreaks” [37]. Recently, Lalchhandama [11] provided a biography of coronaviruses from infectious bronchitis virus (IBV) (discovered in chickens in the early 20th century!) to SARS-CoV-2, “with their evolutionary paradigms and pharmacological challenges”.

## IV. Methods

For configuring the presentation of our research, an underlying citation graph of found SBs has also been constructed through a chain of steps following the metadata lifecycle. These steps involve:

**Conceptualization and Retrieval**. Initially, the required metadata has been listed by a domain conceptualization and observation of the relevant publications by considering topics, authors, years, organizations and keywords. In order to do so, the citation information for articles related to all forms of CoVs which were published between 1965 and 2010 has been collected from Scopus. We impose the constraint that a publication to be an SB must have, at least be sleeping 10 years, thus in our case had to be published before 2010 for being retrieved.

**Acquisition and Integration**. After retrieving the citation information of the papers in CSV format, their bibliographic information was also collected and merged with the citation information file (for later use in drawing collaboration maps in VOSviewer).

**Enrichment and Curation**. After completing the collection of citations, some data cleaning, enrichment, and curation have been applied. The creation of the B-score (discussed in Section IV.2, next) of the so kept articles followed.

### A. Data Collection

For data acquisition and integration, the analyzed data has been collected from the Scopus database on 1 September 2020. We used Scopus due to its wider coverage than WOS. To retrieve data, the following words were used, that is, searched in the title, abstract, and keywords:

“Coronavirus” OR “SARS Coronavirus” OR “Severe Acute Respiratory Syndrome” OR “Middle East Respiratory Syndrome” OR “SARS Virus” OR “Coronaviridae” OR “COVID-19” OR “COVID19” OR “COVID-2019” OR “COVID2019” OR “nCoV-19” OR “nCoV19” OR “nCoV-2019” OR “nCoV2019” OR “2019-nCoV” OR “2019nCoV” OR “MERS-CoV” OR “MERSCoV” OR “MERS Virus” OR “MERS coronavirus” OR “SARS-CoV” OR “SARSCoV” OR “SARS-CoV-2” OR “HCoV NL63” OR “CoV-NL63” OR “HCoVNL63” OR “HCoV-HKU1” OR “CoV-HKU1” OR “HCoVHKU1” OR “HCoV-OC43” OR “CoV-OC43” OR “HCoVOC43” OR “Betacoronavirus” OR “Beta coronavirus” OR “Beta-CoV” OR “BetaCoV” OR “Beta-CoVs” OR “BetaCoVs” OR “Alphacoronavirus” OR “Alpha coronavirus” OR “Alpha-CoV” OR “AlphaCoV” OR “Alpha-CoVs” OR “AlphaCoVs”

### B. Statistical Characteristics

**Beauty Score (B-score).** To find SBs, we use the “beauty score” [38] **(B-score) i**ndex defined as:

\begin{equation*} B=\sum \limits _{t=0}^{t_{m}} \frac {\frac {c_{t_{m}}-c_{0}}{t_{m}}\cdot t+{c}_{0}-c_{t}}{max\left \{{1,c_{t} }\right \}}\end{equation*}

where $t$ is the (discrete integer) year number (referring to the first year of publication ( $t_{0}$) as *0*, the next year *1*, etc., up to $t_{m}$), $c_{t_{m}}$is the number of citations of the considered paper in the last year of interest $t_{m}$ (here, for example, 2020); thus, $c_{0}$ is the number of citations of the considered paper in the publication year $t_{0}$, and $c_{t}$ is the number of citations of the paper in the year called $t$.

The B-score calculates a value corresponding to whether a given paper prototypically represents the SB citation pattern. Thus, papers that gained moderate primary attention, followed by a much greater citation in later years typically receive a relatively high B-score.

We require a paper to have experienced a 10 year sleep period to be considered as an SB; in view of implementing this constraint, we also retrieved the number of citations during the first 10 years after the paper publication. By doing so, we only keep the papers that have an average annual citation of less than 1 in the first 10 years after publication. This approach is gained by hybridizing Ke *et al.* [38] and van Raan’s [39] methods. This approach was also used by Coelho *et al.* [40]. Observe that there is no B-score threshold in order to define and to count an SB; there is only an exclusion principle based on the average number of citations in the first 10 years of the paper existence after its year of publication Thus in this paper, as explained above and following the principles applied in related work, we define a sleeping beauty as all papers that do not violate the exclusion constraint defined above. The importance of a sleeping beauty is determined by its B-score.

Beside the B-score, we also calculate the Relative Specialization Index (RSI) from the Activity Index (AI):

**Relative Specialization Index** (RSI). In order to be representing the level of scientific activity of a country in a field relative to global scientific activity in the same field, one calculates [41].

\begin{equation*} {RSI}_{ij}=\frac {AI_{ij}-1}{AI_{ij}+1}\end{equation*}

in which ${AI}_{ij}$ is

\begin{equation*} {AI}_{ij}=\frac {n_{ij}/n_{i.}}{n_{.j}/n_{..}}=\frac {n_{ij}n_{..}}{n_{i.}n_{.j}}\end{equation*}

where $n_{ij}$ is the number of documents published in the *i-* th field by authors from the $j$-th country; $n_{i.}$ is the number of documents published in the $i$-th field in the world; ${\mathrm { }n}_{.j}$ is the total number of documents for the $j$-th country; and $n_{..}$ is the total number of documents for the whole world.

**Activity Index** (AI). The activity index is an indicator used for systematic comparisons of countries in a given subject. First introduced by Frame [42], it was later developed by Schubert and Braun [43] and Schubert *et al.* [44]. The index indicates relative research efforts of different countries in different subfields or branches of a discipline, and thus represents the emphasis of a country in a particular branch [45]. If ${AI}_{ij}$ is less than 1, this indicates that the $j$-th country’s scientific activity is poor in the $i$-th field, but if ${AI}_{ij}$ is greater than 1, ${AI}_{ij}$ indicates that the $j$-th country’s scientific activity is rich in the $i$-th field.

${RSI}_{ij}$ takes values from −1 to +1. A negative value indicates that the $j$-th country’s scientific activity is poor in the $i$-th field; a positive value indicates that the $j$-th country’s scientific activity is rich in the $i$-th field [41].

Furthermore, VOSViewer [46] was used to draw a cooperation network. Excel and SPSS were used for statistical calculations.

### C. Citation Time Series

To study the evolution of research on Coronavirus Sleeping Beauties (CoVSBs), we arranged all the publications indexed on Scopus in chronological order. The first publication is in 1965, 16 papers on this topic were found in that year. Before that, there is a small number of articles that we did not consider.

We noted down the yearly number of articles published on CoV. Many of the entries are found to be magazine articles, newspaper reports, and blog items which usually do not have any bibliography. In line with our aim of studying the citation history of the CoVSBs, we visually inspected the reference list of each of the articles indexed on Scopus to find out which ones really cite, i.e. refer to CoVSBs.

## V. Results

### A Quick Overview of All Coronavirus Publications

A.

**FIGURE 1.**
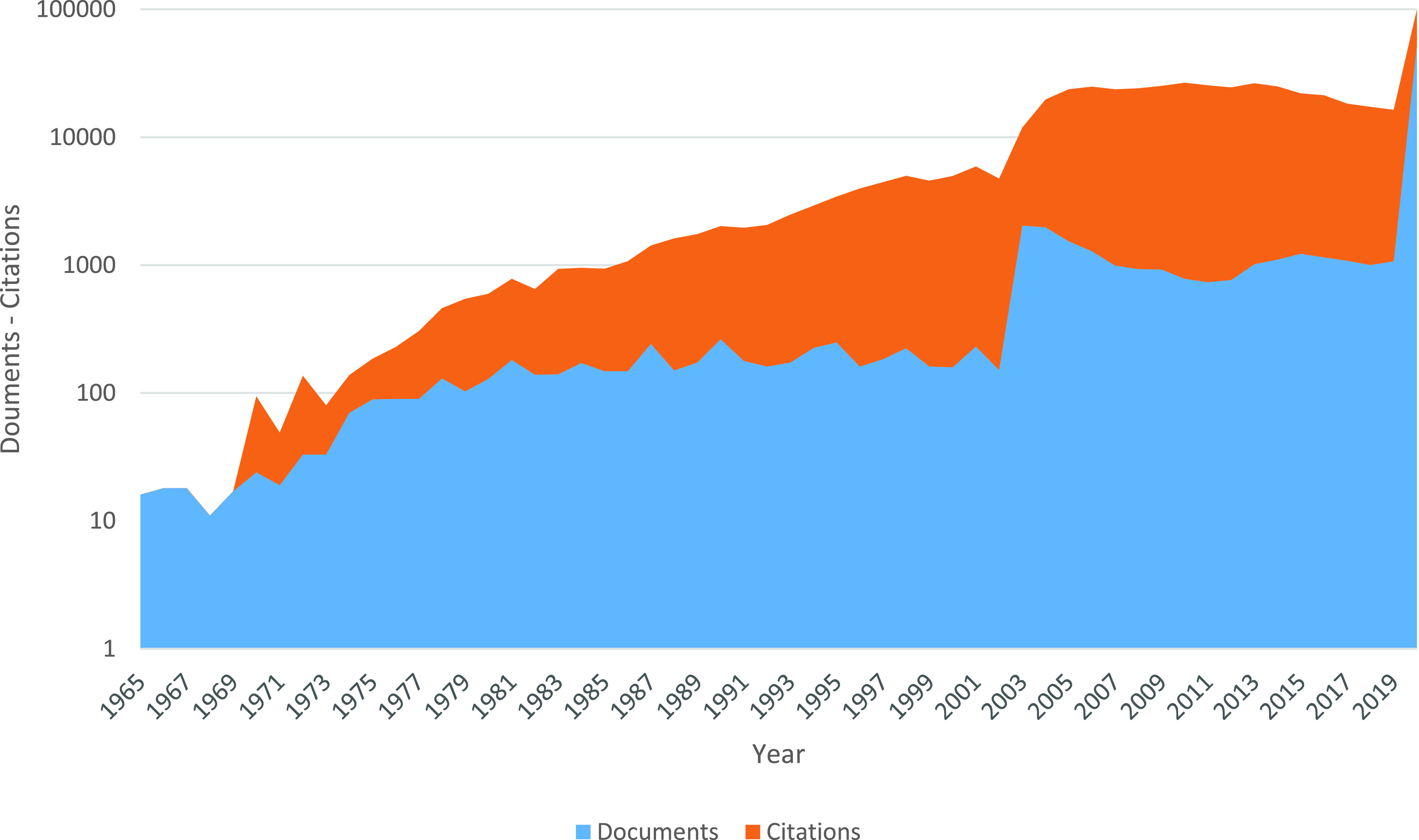
Number of publications and number of their citations in coronavirus research.

This section focuses on the utilization of the gathered data and analysis. In Figure 1, we present the time series which shows that 76956 articles were recorded about coronavirus topics from 1965 till 1 Sept. 2020 (55 years). The highest number of documents (52443) is published in 2020 although this year is not yet over, at the time of writing. The year 2020 includes about 68% of all the documents. Most of these are COVID-19. The result shows that there was also another peak in 2003 with 2038 publication (see the blue part of Figure 1). The red zone in Figure 1 shows the number of citations to previous publications in that year. The process of citing such publications began in 1970 with 70 citations. This number of citations includes citations to 1970 and earlier publications which were calculated in 1970. Citations of Coronavirus publications generally have been steadily increasing. The peaking process of citations has begun in 2003 (one year after the SARS-CoV pandemic). Suddenly the number of citations in 2004 almost doubled as compared to the previous year. It peaked in 2010 and decreased again by 2019. Another huge peak occurs in 2020. Throughout 55 years, 521 papers have not been cited so far (they were published during 1965-1999).

### B. Top SBS in Coronavirus

**FIGURE 2.**
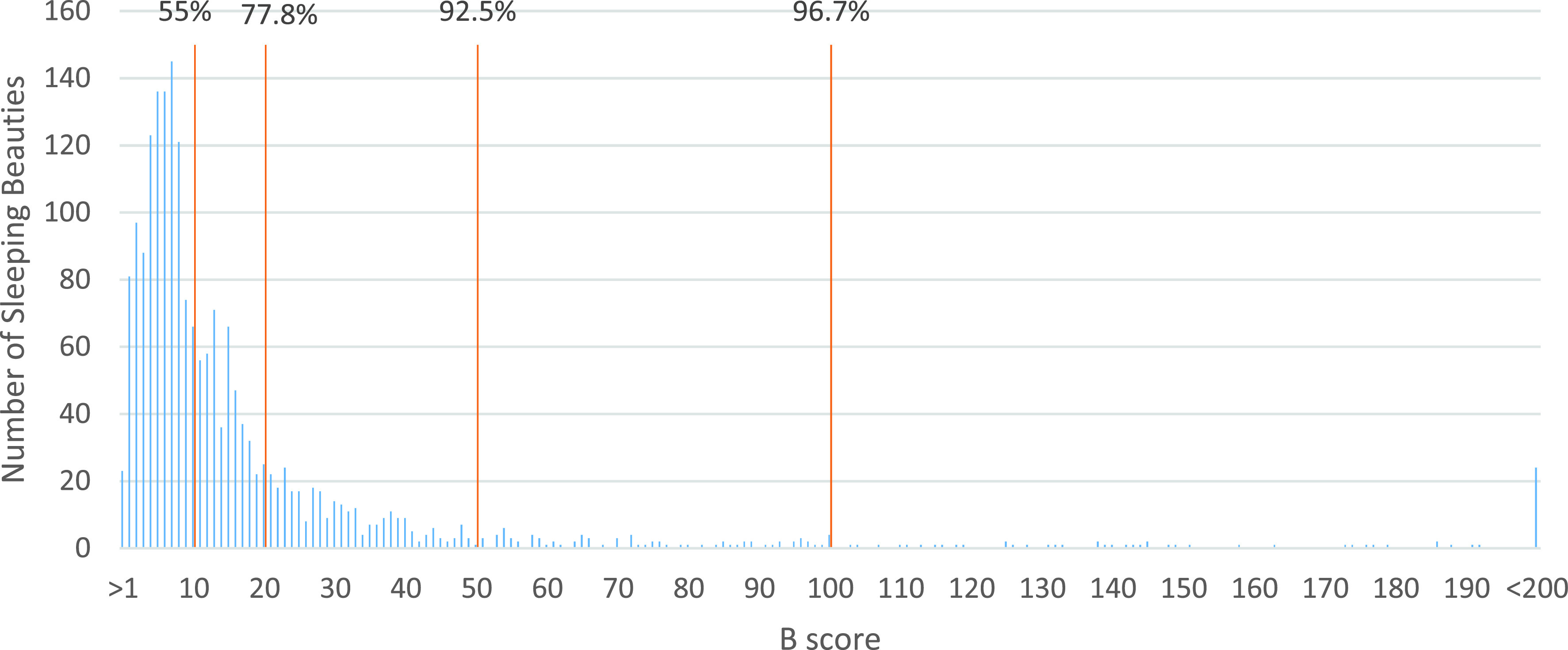
Distribution of all SBs based on B-score (greater than 1 and less than 200).

Figure 2 shows the distribution of SBs based on the B-score. The brown lines indicate the percentile of all SBs with low B-scores. Among the 15363 papers (published between 1965-2010) included in the analysis, we encounter a total of 1979 SB papers among which 148 have a B-score at least equal to 50, and 67 achieve a B-score equal to 100 or greater. As shown in Figure 2, 55% of all SBs have a B-score at least equal to 10, 77.8% have a B-score at least equal to 20, 7.5% of all SBs have a B-score greater than 50, and 3.3% have a B-score greater than 100.

As can be seen in Table 1, the highest B-score belongs to an article which was published in European Journal of Clinical Investigation (i.e., on SARS-coronavirus modulation of myocardial ACE2 expression and inflammation in patients with SARS). This article was published in 2009 and woke up in 2020. This article has been cited 148 times till Sept. 2020. All top 10 Sleeping Beauties with the greatest B-score reach their maximum number of citations in 2020. Furthermore, out of the top 10 Sleeping Beauties, 3 articles had received funds. The top 10 Sleeping Beauties were published between 1990-2010. Also, most of the top Sleeping Beauties are published by authors from China, the United States, and Taiwan. Further content analysis showed that 8 of these 10 papers dealt with “SARS-CoV”.

**TABLE 1:** Top 10 Sleeping Beauties on Coronavirus: Overall

Paper ID	Title	Authors	Journal	Collaborator Countries	Funded Yes/No	Year of Publication	Year of Maximum Citations	Total citations	Annual citation (for the first 10 years for publication.)	Annual citation (Overall)	B-score
A1	SARS-coronavirus modulation of myocardial ACE2 expression and inflammation in patients with SARS	Oudit et al. [47]	European Journal of Clinical Investigation	Canada, China, Austria	Yes	2009	2020	148	0.8	2.6	800.7
A2	Plasma glucose levels and diabetes are independent predictors for mortality and morbidity in patients with SARS	Yang et al. [48]	Diabetic Medicine	China, US, Hong Kong	No	2006	2020	117	0.7	2	780.8
A3	Binding of SARS coronavirus to its receptor damages islets and causes acute diabetes	Yang et al. [49]	Acta Diabetologica	China	Yes	2010	2020	114	0.5	2	589.7
A4	Safe tracheostomy for patients with severe acute respiratory syndrome	Wei et al. [50]	Laryngoscope	Hong Kong	No	2003	2020	58	0.5	1	463
A5	Cardiovascular complications of severe acute respiratory syndrome	Yu et al. [51]	Postgraduate Medical Journal	Hong Kong	No	2006	2020	61	0.3	1	432
A6	Neurological manifestations in severe acute respiratory syndrome	Tsai et al. [52]	Acta Neurologica Taiwanica	Taiwan	No	2005	2020	54	0.1	0.9	405
A7	Air pollution and case fatality of SARS in the People’s Republic of China: An ecologic study	Cui et al. [53]	Environmental Health: A Global Access Science Source	China	Yes	2003	2020	56	0.9	1	395.1
A8	The time course of the immune response to experimental coronavirus infection of man	Callow et al. [54]	Epidemiology and Infection	UK	No	1990	2020	77	0.3	1.3	352.2
A9	Cerebrospinal fluid antibodies to coronavirus in patients with Parkinson’s disease	Fazzini et al. [55]	Movement Disorders	US	No	1992	2020	52	0.7	0.9	332.3
A10	Olfactory neuropathy in severe acute respiratory syndrome: Report of a case	Hwang [56]	Acta Neurologica Taiwanica	Taiwan	No	2006	2020	42	0	0.7	306.5

Note that the publication numbers shown in Table 1 were obtained and are ranked by sorting their B-score (high to low); since no limits were imposed on the collection, we call these papers the “Overall SBs” set.

**FIGURE 3.**
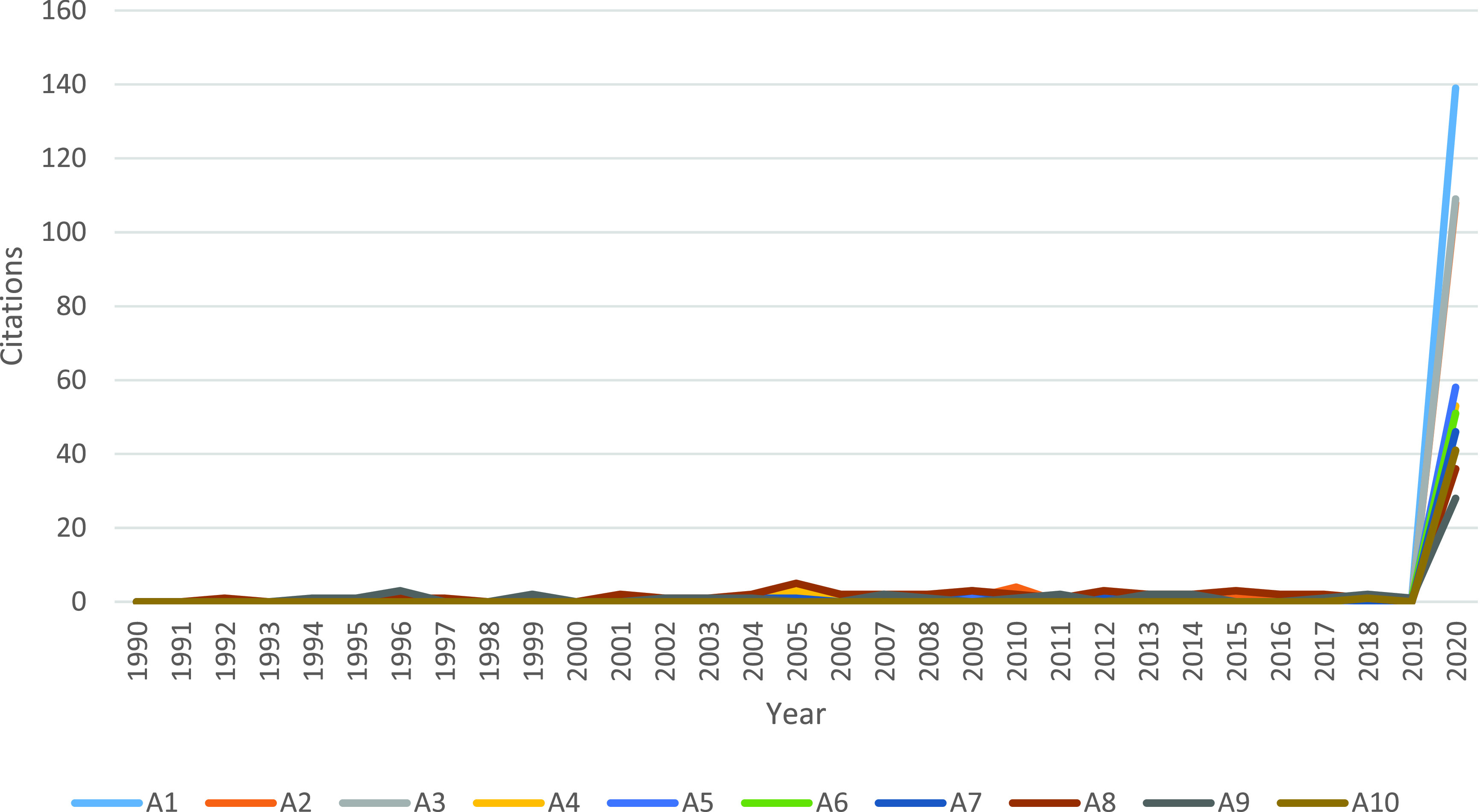
The number of citations of the top 10 SBs (“A” papers in table 1).

Figure 3 shows the citation trend of the top 10 SBs mentioned in Table 1. These top 10 “Overall SBs” reached their highest number of citations in 2020, simultaneously with the spread of the COVID-19 pandemic. In general, these 10 SBs received between 28 and 139 citations in the year of awakening (2020). The A10 paper got the lowest annual number of citations in the first 10 years of publication (in fact no citation), in contrast to the A7 which has the greatest annual citation number in the first 10 years of publication (0.9 citation per year). The A1 paper received only 9 citations during the years 2009-2019 and suddenly received 139 citations in 2020. Even with a low number of citations during the 2009-2019 period, but receiving a high number of citations in 2020 A1 is a high B-score paper.

Table 2 shows the top 10 Sleeping Beauties published before 2000. By selecting such a sample, we show that for elderly SBs at least 20 years have passed since their publication. The duration between the publishing date and the awakening of these papers ranges between 28 and 52 years. As Table 2 suggests, the Sleeping Beauties (in Table 2) are completely different from those in Table 1. Also, the first Sleeping Beauties in Table 1 and Table 2 have significantly different B-scores. Like for the SBs in Table 1, we noticed that only 3 out of 10 received funds. The oldest SB (B3) in Table 2 is a short note published in Nature in 1968. Of course, the total number of citations is not high, but, – because the paper has been asleep for a very long time, it is one of the SBs with a high B-score. Paper B4, has the most citations among the papers in this table. Four of these 10 papers have been published by authors affiliated with institutions in the US and UK; the rest of these 10 was published by authors affiliated with institutions in various countries. The lowest annual citation number for the first 10 years belongs to the B3 paper while the greatest belongs to B5.

**TABLE 2:** Top 10 Sleeping Beauties on Coronavirus: Published Before 2000

Paper ID	Title	Authors	Journal	Collaborator Countries	Funded Yes/No	Year of Publication	Year of Maximum Citations	Total citations	Annual citation (for the first 10 years for publication)	Annual citation (Overall)	B-score
B1	The time course of the immune response to experimental coronavirus infection of man	Callow et al. [54]	Epidemiology and Infection	UK	No	1990	2020	77	0.3	1.3	352.2
B2	Cerebrospinal fluid antibodies to coronavirus in patients with Parkinson’s disease	Fazzini et al. [55]	Movement Disorders	US	No	1992	2020	52	0.7	0.9	332.3
B3	Virology: Coronaviruses	No author name available [57]	Nature	Unknown	No	1968	2020	13	0	0.2	304.7
B4	Survival characteristics of airborne human coronavirus 229 $^{\mathbf {E}}$	Ijaz et al. [58]	Journal of General Virology	Canada	Yes	1985	2020	81	0.5	1.4	205.7
B5	Antibodies to coronaviruses OC43 and 229E in multiple sclerosis patients	Salmi et al. [59]	Neurology	Finland	No	1982	2020	49	0.8	0.8	192.4
B6	The effect of ascorbic acid on infection of chick-embryo ciliated tracheal organ cultures by coronavirus	Atherton et al. [60]	Archives of Virology	Australia	No	1978	2020	15	0.1	0.2	185.8
B7	Medical reviews. Coronaviruses	Monto [61]	Yale Journal of Biology and Medicine	US	No	1974	2020	31	0.4	0.5	179.4
B8	Epidemiology of coronavirus respiratory infections	Isaacs et al. [62]	Archives of Disease in Childhood	UK	Yes	1983	2020	50	0.6	0.8	177.3
B9	Immunofluorescence studies on the pathogenesis of hemagglutinating encephalomyelitis virus infection in pigs after oronasal inoculation	Andries and Pensaert [63]	American Journal of Veterinary Research	Belgium	No	1980	2020	41	0.4	0.7	173.1
B10	Virucidal efficacy of physico-chemical treatments against coronaviruses and parvoviruses of laboratory animals	Saknimit et al. [64]	Jikken dobutsu. Experimental animals	Japan	Yes	1988	2020	25	0.2	0.4	163.3

Further content analysis showed that 9 of these 10 papers generally dealt with “infection” and “antibody”; 4 of these specifically mention the “Human coronavirus 229E”.

**FIGURE 4.**
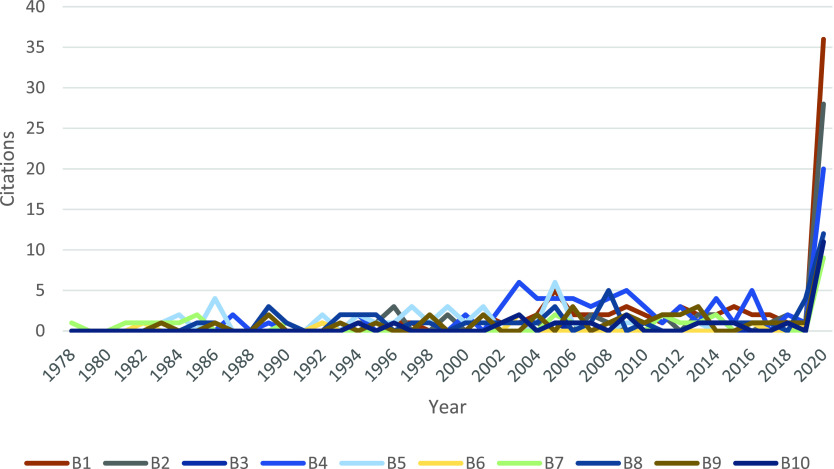
The number of citations of top 10 SBs on coronavirus (“B” papers in table 2).

Figure 4 suggests that the top 10 Sleeping Beauties published before 2000 have started to receive a high number of citations in 2020. Note that none of these articles received citations before 1978. Figure 4 also shows that the B4 article published in the Journal of General Virology has experienced a minor peak in 2003; B5 also has a peak in 2005 (both with 6 citations). This peak could be due to the SARS outbreak during 2002–2004. Generally, after minor ups and downs, all of these 10 papers awoke in 2020.

### C. Publication Year and Citation Year of SBS

**FIGURE 5.**
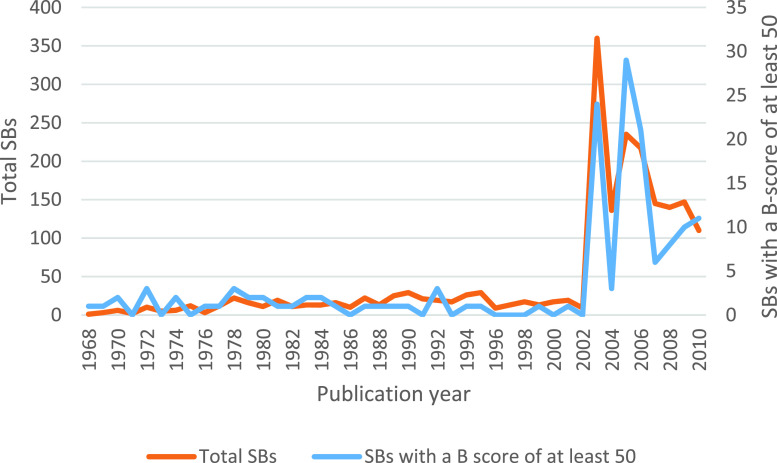
Number of SBs publications per year distinguishing two groups: all the BSs and those with a B-score higher than 50.

Figure 5 shows the number of SBs published during each year. The brown line shows the number of overall SBs but the blue one shows the number of SBs with a B-score of at least 50. These two groups almost have a similar publication trend. In both groups, most of the papers have been published in 2003 and 2005.

**FIGURE 6.**
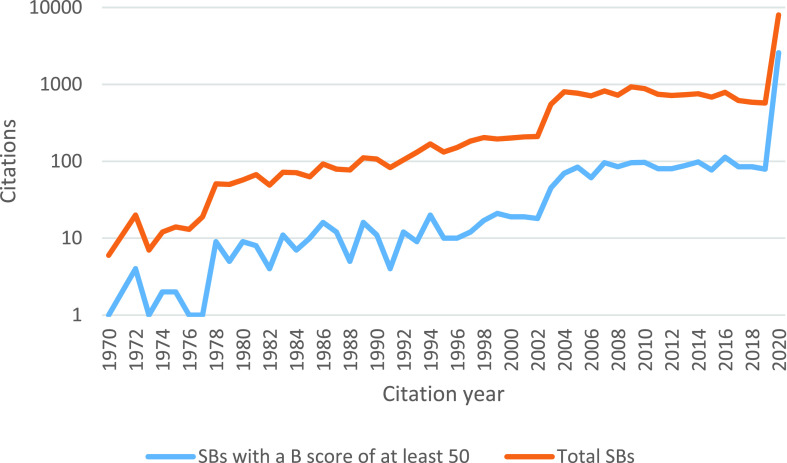
Number of SBs citations per year, distinguishing two groups as explained in the text.

Figure 6 reports the sum of SB citations for each year. This figure also shows two groups of SBs. The brown line shows the citation amount of the so-called overall SBs, while the blue line shows the citation number of SBs with a (high) B-score, - being at least 50. Both groups received the greatest number of citations in 2020. The citation number evolution for these groups is rather similar.

### D. Top Countries, Institutions, Authors, and Journals With Most SBS

Table 3 shows the top 10 countries with the most SBs distinguishing two groups as before. The first group is based on the overall (1979) SBs, the second part is based on the (148) SBs with a B-score of at least 50.

**TABLE 3:** Top 10 Countries With Most SB Papers

Based on the overall Sbs set				Based on the SBs set, those with a B-score of at least 50			
Country name	Number of SBs	Total number of papers in Coronavirus (1965 - 1 Sept. 2020)	Relative Specialization Index (RSI)	Country name	Number of SBs	Total number of papers in Coronavirus (1965 - 1 Sept. 2020)	Relative Specialization Index (RSI)
US	414	20132	0.009	China	40	10110	0.151
China	342	10110	0.151	Hong Kong	24	1967	0.672
Hong Kong	216	1967	0.672	US	18	20132	0.009
Canada	149	3580	0.155	Canada	16	3580	0.155
UK	112	7511	0.143	Singapore	13	1401	0.586
Taiwan	105	1186	0.194	Taiwan	12	1186	0.194
Japan	89	1756	−0.419	Australia	7	2512	0.120
Singapore	84	1401	0.586	Japan	7	1756	−0.419
Germany	66	3370	−0.169	UK	5	7511	0.143
France	57	3132	0.007	France	4	3132	0.007

In the first group, i.e., all SBs whatever their B-score, most of the SB papers are published by authors affiliated with US institutions. Also, this “country” published the greatest number of papers on the Coronavirus topic (until Sept. 01, 2020). Hong Kong has the highest Relative Specialization Index (0.672) in the Coronavirus topic (of course between the countries named Table 3).

In the second group, China has the highest number of SBs with such a B-score of at least 50 (40 SBs); Hong Kong takes second place (24 SBs), and the US the third place (18 SBs). One knows that Hong Kong is a Chinese special administrative region and we distinguish it when observing data in both groups; if we merge Hong Kong data to that of China, China moves up to the first place with a significant difference (= 64 SBs, of course), with respect to the next follower, USA.

**FIGURE 7.**
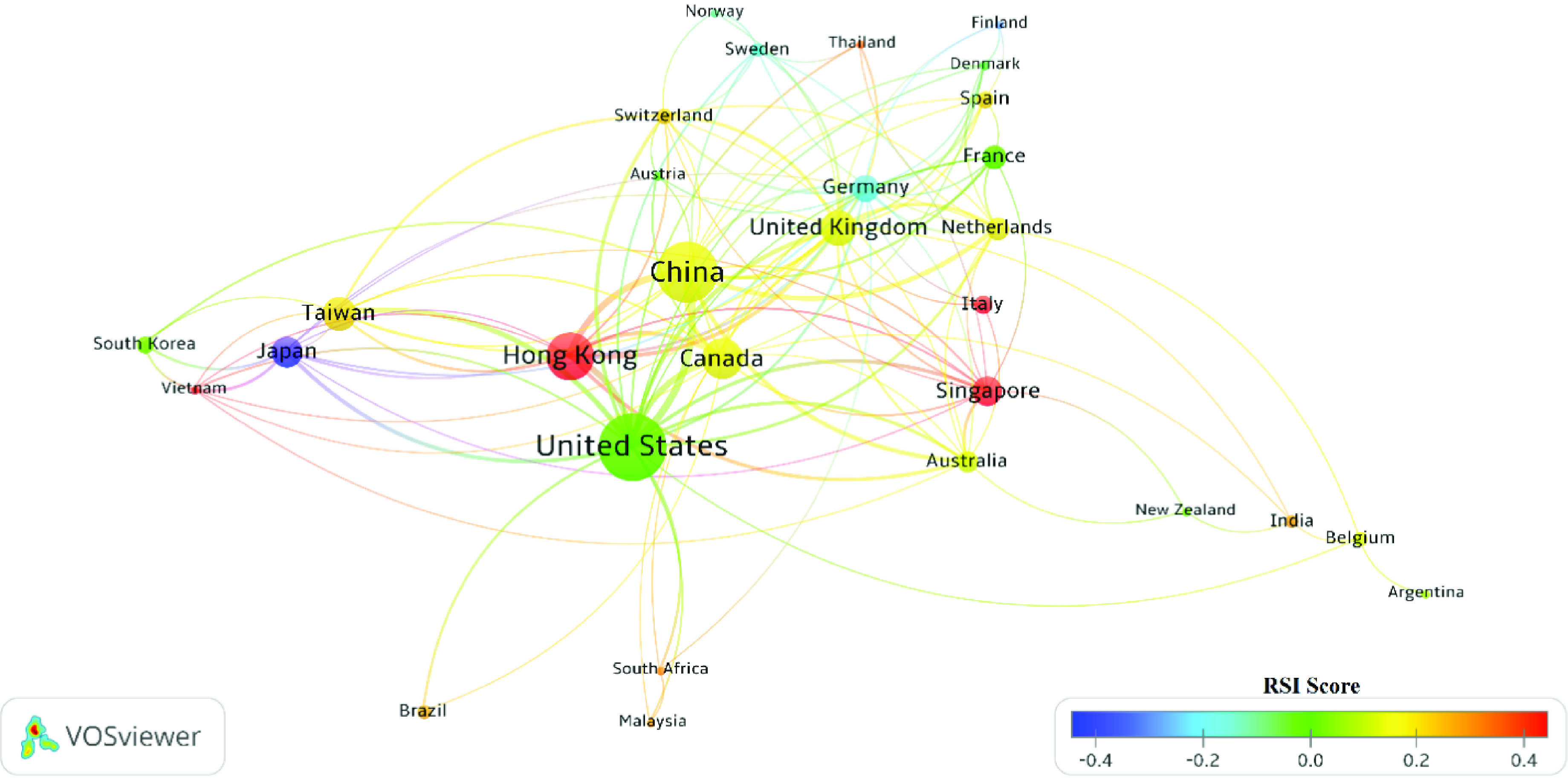
Cooperation network of countries with the largest number of SB papers (based on all SBs group).

Figure 7 shows the international collaboration map of countries having SBs (based on the overall SBs set) A particular node size indicates the number of SB papers; the thickness of the edges indicates the number of collaboration; the node colors show the RSI score of the country. As observed in Table 3, the US, China, and Hong Kong have the most SBs, respectively. The United States and China (23 shared papers) have collaborated intensively. Hong Kong (0.672), Singapore (0.586), and Vietnam (0.509) have the highest RSI score in the Coronavirus topic, respectively. Most Asian countries in this network have a high RSI value.

Table 4 shows the top 10 institutions with the largest number of SBs allowing some emphasis on “differences” between two distinguished groups. The first group contains the journals that published the greatest number of SBs (based on overall SBs); the second, those with the SBs having a B-score of at least 50. The Chinese University of Hong Kong has first place in both groups. Most of these top 10 institutions belong to China and Hong Kong. The authors affiliated with the University of Hong Kong published most of the SB papers. Generally, these 10 institutions in the second group published about 38.5% of SBs with a B-score of at least 50; about 22% of the overall SBs have been published by the first section institutions.

**TABLE 4:** Top 10 SB Papers Authors

Based on the overall SBs set			Based on SBs with a B-score of at least 50		
Institution name	Country	Number of SBs	Institution name	Country	Number of SBs
Chinese University of Hong Kong	Hong Kong	85	Chinese University of Hong Kong	Hong Kong	9
The University of Hong Kong	Hong Kong	81	Tan Tock Seng Hospital	Singapore	8
Prince of Wales Hospital Hong Kong	Hong Kong	53	University of Toronto	Canada	7
University of Toronto	Canada	40	Prince of Wales Hospital Hong Kong	Hong Kong	6
Chinese Academy of Sciences	China	35	Princess Margaret Hospital Hong Kong	Hong Kong	5
Tan Tock Seng Hospital	Singapore	32	Capital Medical University	China	5
Queen Mary Hospital Hong Kong	Hong Kong	30	Academy of Military Medical Science China	China	5
National University of Singapore	Singapore	27	The University of Hong Kong	Hong Kong	4
Chinese Academy of Medical Sciences and Peking Union Medical College	China	27	National University of Singapore	Singapore	4
Centers for Disease Control and Prevention	USA	25	National Taiwan University Hospital	Taiwan	4

The authors with most SBs are also divided into “Based on overall SBs” and “Based on SBs with a B-score of at least 50”. The first ranked author, based on his total number of SBs, is Joseph Jao Yiu Sung affiliated with the Chinese University of Hong Kong, Hong Kong. Most of the top 10 authors with most SBs with a B-score of at least 50 are affiliated with China’s institutions (Table 5). Of course, these authors have published a handful of SBs.

**TABLE 5:** Top 10 of Authors With Most SB Papers

Based on overall SBs			Based on SBs with a B-score of at least 50		
Author name	Affiliation	Number of SBs	Author name	Affiliation	Number of SBs
Joseph Jao Yiu Sung	Chinese University of Hong Kong, Hong Kong	19	Min Jin	Tianjin Institute of Environmental and Operational Medicine, China	4
Paul KS Chan	Chinese University of Hong Kong, Hong Kong	14	Chao Liu	Beijing Institute of Pharmacology and Toxicology, China	4
Fumihiro Taguchi	National Institute of Infectious Diseases, Japan	13	Nong Song	Tianjin Institute of Environmental and Operational Medicine, China	4
Marian C. Horzinek	Utrecht University, Netherlands	11	Wei Wei	Academy of Military Medical Science China, China	4
Gabriel M. Leung	The University of Hong Kong, Hong Kong	11	Jing Yin	Tianjin Institute of Environmental and Operational Medicine, China	4
Jane Parry	Hong Kong, France	11	Bei Zhen	Academy of Military Medical Science China, China	4
Kwok Yung Yuen	The University of Hong Kong, Hong Kong	11	Fuhuan Chao	Tianjin Institute of Hygienic and Environmental Medicinal Science, China	3
Chang, S.C.	National Taiwan University Hospital, Taiwan	11	Tongyu Fang	Beijing Institute of Basic Medicine, China	3
Ralph S. Baric	The University of North Carolina at Chapel Hill, USA	10	Qingxin Kong	Key Laboratory of Risk Assessment and Control for Environment & Food Safety, China	3
Luis Enjuanes	CSIC - Centro Nacional de Biotecnologia (CNB), Spain	10	Jinsong Li	Academy of Military Medical Science China, China	3

**TABLE 6:** Top 10 Journals With the Largest Number of SB Papers

Based on the overall Sbs set			Based on the SBs set, Sbs with a B-score of at least 50		
Journal name	Number of SBs	IF (2019)	Journal name	Number of SBs	IF (2019)
Advances in Experimental Medicine and Biology	84	2.450	Archives of Virology	5	2.243
Archives of Virology	35	2.243	Advances in Experimental Medicine and Biology	3	2.450
Lancet	35	60.39	Annals Academy of Medicine Singapore	3	1.533
Hong Kong Medical Journal	29	1.679	Journal of Infectious Diseases	3	5.022
Journal of General Virology	25	3.376	Journal of Medical Virology	3	2.021
American Journal of Veterinary Research	21	0.811	Acta Neurologica Taiwanica	2	–
Journal of Virology	21	4.501	American Journal of Veterinary Research	2	0.811
Virology	20	2.819	Canadian Journal of Surgery	2	1.610
Emerging Infectious Diseases	19	6.259	Epidemiology and Infection	2	2.152
Nature	19	42.77	European Journal of Clinical Microbiology and Infectious Diseases	2	2.837

Like before, also the top 10 journals that published the greatest number of SBs are divided into two groups. The first group contains the journals that published the greatest number of overall SBs. The greatest number of SBs has been published by “Advances in Experimental Medicine and Biology”. About 15.5% of the total overall SBs have been published in these journals. Lancet and Nature, two famous journals, can be observed among these journals. In the second group, one shows the journals that published the greatest number of “SBs with a B-score of at least 50”. The greatest number of SBs has been published by “Archives of Virology”. Among these journals, “Journal of Infectious Diseases” has the highest Impact Factor.

### E. Most Frequent Keywords in SB Publications

**FIGURE 8.**
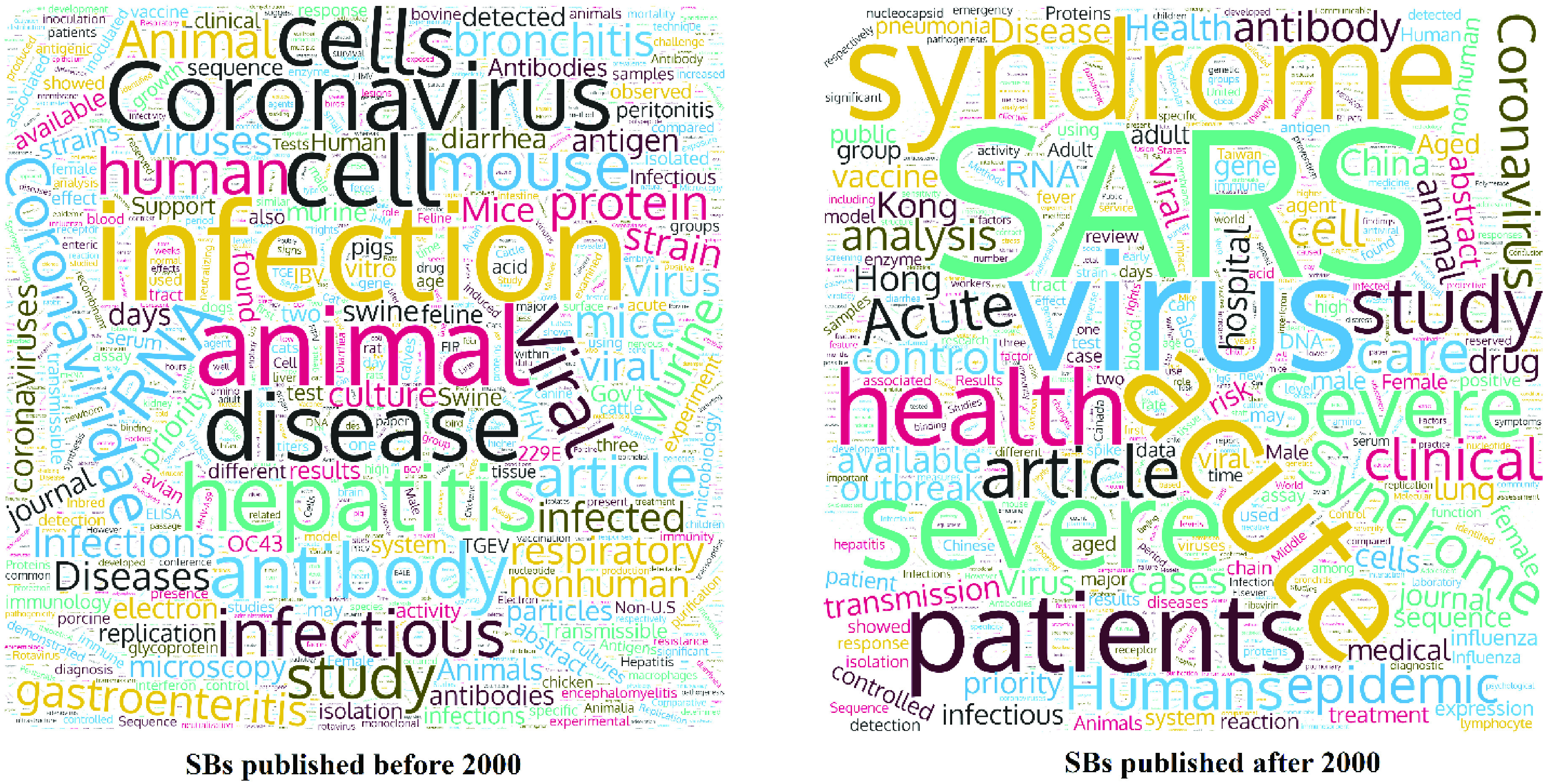
Sleeping beauties word cloud (based on overall SBs).

Figure 8 shows the main words extracted from the title, abstract, author keywords, and indexed keywords of SBs (based on overall SBs). The “word cloud” on Figure 8 left-hand side shows the frequent SBs keywords for papers that have been published before 2000. As can be seen, “Infection” and “Animal” are the most frequent words there; the “word cloud” on the right-hand side of Figure 8 shows the most frequent SBs keywords for papers that have been published after 2000. “Virus” and “Severe Acute Respiratory Syndrome (SARS)” were the most common keywords. Actually, among the 1979 SB papers, 1151 were related to SARS-CoV; among the 148 SB papers with a B-score greater than 50, 105 were related to SARS-CoV. This proves that more than half of SBs have dealt specifically with the SARS-CoV, among the various CoVs.

## VI. Discussion and Challenges

### A. Discussion

The empirical evaluations show that there are two major peaks in the publishing rate of coronavirus (CoV)-related research works: one in 2003, the other in 2020. Both of these jumps in the publishing records –for which 68% of the whole publications in our sample occurs in 2020 –, are related to the present pandemic, one for SARS in 2002, and the other for COVID in 2019. Our analysis shows that a high number of SBs are papers of authors in institutions located in China and Hong Kong which are also the regions from which the viruses in both cases are thought to have been early identified. This also may explain the reason why Singapore and Taiwan are among the top 10 producers of SBs, even though usually these countries are not shown in many of the lists for other driving research topics. The presence of such countries is also visible among the magazines and journals which are usually considered to be the top ones in the respective fields, namely Science, Nature, and Lancet. Most of the authors hold affiliations from China, - which also may explain the local plethora of investigations on SARS and/or COVID research. The visualization of the keywords also shows that more than half of SBs are corresponding to SARS papers which have awoken in 2020. These facts reveal a strong connection between SARS and COVID-19 which lied in SBs for years and are only awakened after the happening of the second pandemic.

The conceptual and bibliographic disjointness of our work and other related publications lies in the research questions. Thus, we point out the main presently added values of this research. While many of the recent works [12]–[16] have either constructed a collection of structured publications information or provided statistical analysis over the COVID-19 publications, we focus on the historical data and the role of sleeping beauties in this particular topic. In [12], a comprehensive report about the research activities of COVID-19 is provided by looking into the relevant publications. However, the overview of SBs and the impact of the global pandemic in their awakening is overlooked. The same problem is visible in [16]. In our studies, we shine lights on the already existing SBs which are highly important research works, awakened in 2003 and 2020. We highlight the top stakeholders including countries, journals and magazines, authors, and their affiliations, as well as the research keywords. Our findings cover the here above-defined research questions.

In response to the first research question, observed in our results, one can see that there has been already effective research about coronaviruses in the previous decade. However, most of those works were in a sleeping status due to low attention from the relevant communities. While there has been an expectation of a global pandemic happening due to this type of viruses, and despite the given alarms around this topic, the related research remained without much attention.

The hype of investigation and awakening of SBs in coronavirus research only happened in the COVID-19 initial pandemic year, 2020 according to the World Health Organization (WHO), on March 11, 2020; see: https://www.who.int/director-general/speeches/detail/who-director-general-s-opening-remarks-at-the-media-briefing-on-covid-19-11-march-2020

In answering the second research question, looking at the names of the active countries as well as the funding bodies, we conclude that the high attention paid by certain countries relates to their probable vulnerabilities in facing the virus as well as having strong research facilities. It is also visible that those countries such as the UK, China, and the USA have been the origination point of most SBs.

We have also identified the most relevant journals in which related articles to coronavirus and their corresponding SBs have been published.

Overall, the evaluations show that the effectiveness of investigations is the infrastructure of the science, besides the research itself in which the SBs of different research topics stay awakened.

## VII. Conclusion and Future Vision

The present work may be a primary step towards creating an inclusive model, as demanded or suggested [65], with a direct influence of research results toward societies resilient towards not only global pandemics, but also hazards and disasters. Ignoring significant attention to certain research results for years and not having a systematic analysis of individual results lead to a situation in which research results are sleeping for a while and are only partially awakened, - even somewhat accidentally [9], [23], [32], or as in the present case, in global panics. The damages this brings in all levels of personal, local, and global are alas irreparable.

The results of our study give some initial evidence that more attention on the sleeping beauties of SARS and COVID research would have put the current research about COVID-19 in a much better situation. Advanced analysis on such topics in science and structured representation of them including the FAIR data principles [66], can serve researchers in some discovery of early responses for global panics if not preventing them completely. Our vision is an inclusive search platform where research results are active and awakened since their existence. Having a certain analysis at hand, one can immediately access the funding bodies, stakeholders, and main players of any topic, especially when it is about global health and the survival of millions of people. Topic-based research movements also give an immediate historical analysis of problems and challenges where a certain amount of research has already been conducted. All of these provide shortcuts for solutions and save months of indirect research when seconds matter for life rescue.
